# Fine-Grained Image Recognition with Bio-Inspired Gradient-Aware Attention

**DOI:** 10.3390/biomimetics10120834

**Published:** 2025-12-12

**Authors:** Bing Ma, Junyi Li, Zhengbei Jin, Wei Zhang, Xiaohui Song, Beibei Jin

**Affiliations:** 1 Institute of Physics, Henan Academy of Sciences, Zhengzhou 450046, China; 2 Henan Academy of Sciences, Zhengzhou 450046, China; 3 College of Electronic and Electrical Engineering, Henan Normal University, Xinxiang 453007, China; 4 School of Medicine, Sun Yat-sen University, Shenzhen 518107, China; 5 School of Physics, Henan Normal University, Xinxiang 453007, China

**Keywords:** computer vision, image recognition, attention mechanism, vision transformer

## Abstract

Fine-grained image recognition is one of the key tasks in the field of computer vision. However, due to subtle inter-class differences and significant intra-class differences, it still faces severe challenges. Conventional approaches often struggle with background interference and feature degradation. To address these issues, we draw inspiration from the human visual system, which adeptly focuses on discriminative regions, to propose a bio-inspired gradient-aware attention mechanism. Our method explicitly models gradient information to guide the attention, mimicking biological edge sensitivity, thereby enhancing the discrimination between global structures and local details. Experiments on the CUB-200-2011, iNaturalist2018, nabbirds and Stanford Cars datasets demonstrated the superiority of our method, achieving Top-1 accuracy rates of 92.9%, 90.5%, 93.1% and 95.1%, respectively.

## 1. Introduction

**Figure 1:**
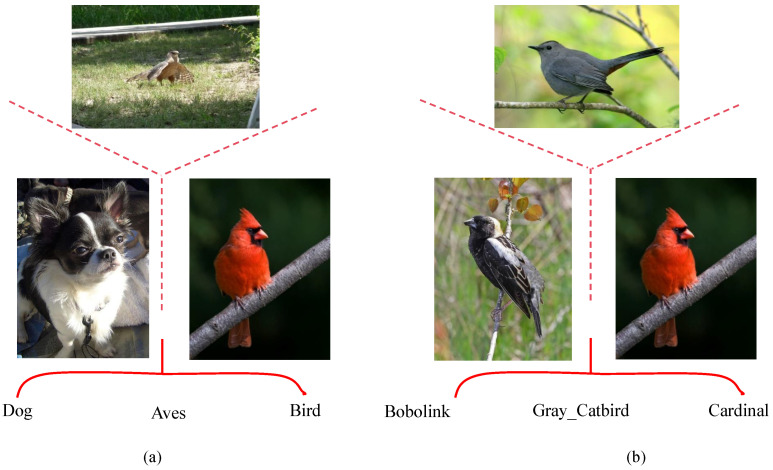
Illustration of (**a**) coarse-grained classification, where inter-class differences are large and easy to separate, and (**b**) fine-grained classification, where categories differ only in subtle local attributes.

Fine-grained image recognition is vital for applications ranging from ecological monitoring to intelligent retail. As shown in Figure 1, it involves discriminating between subordinate categories under the same superordinate class (e.g., bird species) and is characterized by two principal challenges: inter-class differences can be extremely subtle, relying on fine local cues like edges and textures, while intra-class variations can be substantial due to factors such as pose, occlusion, and illumination, making it difficult to learn representations that are both discriminative and robust.

Recent research on fine-grained visual recognition has mainly developed in two directions. The first one emphasizes the positioning and cropping of differentiated areas. These methods typically rely on rigid rectangular proposals to detect and crop protruding areas [1]. However, such strategies often introduce background noise and fail to capture structural relationships among regions, leading to overly large or imprecise bounding boxes. The second one leverages attention mechanisms to extract discriminative information directly from the full image. For instance, TransFG [2] employs counterfactual attention to suppress background interference and improve region localization. MetaFormer [3] enriches discriminative semantics by injecting auxiliary meta-information—such as geographical, attribute, or textual cues—into visual tokens. Dual-Fusion [4] enhances representations through cross-layer feature fusion without heavy annotation overhead. MPSA [5] introduces partial sampling attention for multi-scale representation learning, while HERBS [6] incorporates high-temperature refinement and background suppression modules to strengthen discriminative features and reduce background noise in multi-granularity learning.

Despite these advances in modeling discriminative regions, two underlying issues remain largely unaddressed. The progressive feature aggregation in Transformer architectures tends to weaken high-frequency components such as edge and texture gradients, causing a loss of fine local details that are critical for discriminating highly similar categories; Although discriminative regions can be localized, there is a lack of explicit modeling for continuously and reliably incorporating these high-frequency structural cues into the global representation. This missing systematic propagation pathway restricts the model’s capacity to achieve an optimal balance between discriminability and robustness.

Research [7] has demonstrated that in the hierarchical processing of the human visual cortex, gradients serve as fundamental and powerful low-level visual features, while the attention mechanism optimizes information processing by enhancing neural signals associated with task-relevant gradient information. In the classic theory of computational neuroscience and cognitive science, the Feature Integration Theory [8], visual processing is divided into two stages. In the preattentive stage, the visual system processes basic features of the entire scene in parallel and automatically, such as color, orientation, motion, and spatial frequency (the basis of gradients). These features are registered in specific “feature maps.” In the feature integration stage, attention binds these disparate features together to form the objects we perceive. Edges and contours in an image correspond precisely to areas of high activity in the orientation feature maps. These high-gradient regions generate strong signals during the preattentive stage and become candidate foci for attention.

We draw inspiration from the above theory and propose a gradient-aware spatial attention framework for fine-grained image recognition. Our core idea is to directionally recalibrate spatial attention using explicit gradient information and encode the fine gradient representation into a compact gradient token, which is then injected into the Transformer. This makes it possible to jointly model local high-frequency details and global semantics. The main contributions are:

1. We propose an efficient method for constructing gradient-aware spatial weight maps, which effectively encodes high-frequency direction cues at an extremely low computational cost through multi-directional one-step attention fusion.
2. We propose a strategy of injecting compact gradient tokens into the Transformer, which enables the model to explicitly utilize high-frequency local information while maintaining the global semantic modeling capability.
3. The proposed method is evaluated on four widely used fine-grained benchmarks: CUB-200-2011, iNaturalist 2018, NABirds, and Stanford Cars, achieving top-1 accuracies of 92.9%, 90.5%, 93.1%, and 95.1%, respectively, demonstrating its effectiveness.

## 2. Related Work

### 2.1. Fine-Grained Image Recognition

Fine-grained image recognition addresses the modeling problem of subtle appearance differences between homologous categories, with the aim of accurately distinguishing visually similar subordinate categories.

Early approaches [9,10,11] typically relied on strongly supervised annotations such as part keypoints, bounding boxes, or segmentation masks to guide models toward discriminative regions. While effective at enhancing local feature discriminability, these methods suffer from high annotation cost and poor scalability. Subsequent research [12,13,14] therefore explored self-supervised, weakly supervised, or structured network designs that aim to automatically discover discriminative parts and strengthen local representations. In addition, auxiliary modalities such as textual cues or spatio-temporal/geographic priors [15] have been leveraged to enrich representations and suppress background noise. Although these auxiliary modalities can improve robustness, they depend on the availability and reliability of external sources, and effectively incorporating such priors while preserving generalization remains challenging.

In fine-grained image recognition, discriminative features between different categories often reside in extremely subtle local differences. Introducing explicit priors, such as shape structure, object contours, and multi-scale texture patterns, has become a key strategy for enhancing model discriminability. Khan et al. [16] propose a Region of Interest (ROI)-based object recognition model to address limitations in existing methods by efficiently identifying objects at any image location while handling challenges like overlapping objects and noisy backgrounds. Cross-X [17] facilitates multi-scale feature correspondence by establishing associations across different images and network layers, explicitly encouraging the model to capture discriminative cues ranging from local details to global patterns. Han et al. [18] propose an end-to-end Part-based Convolutional Neural Network (P-CNN) that integrates channel attention, automatic part localization, and multi-stream classification with a novel Duplex Focal Loss to achieve effective fine-grained visual categorization. These methods, by embedding strong priors closely aligned with task requirements, perform exceptionally well in scenarios with limited data or well-defined prior knowledge. However, their performance heavily depends on the quality and completeness of the incorporated priors, which, to some extent, constrains their generalization capability.

### 2.2. Attention Mechanism

The core idea of the attention mechanism is to enable models to dynamically focus on the most important parts of the input. Its development originated from sequence-to-sequence learning in machine translation, initially manifested as the additive attention proposed by Bahdanau et al. [19] and the multiplicative attention introduced by Luong et al. [20]. These early mechanisms computed association weights between hidden states to achieve alignment of the source sequence, shifting information processing from static encoding to dynamic focusing and laying the groundwork for handling long sequences and complex dependencies. Subsequently, Vaswani et al. [15] pioneered the Transformer architecture in 2017, which relied entirely on the self-attention mechanism and completely abandoned recurrent and convolutional structures. The core of this architecture—scaled dot-product attention—leverages the query–key–value model to compute global dependencies in parallel, significantly enhancing training efficiency and long-range modeling capability. The success of Transformer established attention as a fundamental component of deep learning and catalyzed the wave of pre-trained models such as BERT [10] and GPT [21].

Ref. [22] first applied the standard Transformer directly to image recognition, introducing the Vision Transformer (ViT), which achieves performance comparable to or exceeding that of CNNs on multiple benchmarks. Relative to CNNs, ViT imposes fewer image-specific inductive biases and therefore offers greater modeling flexibility, but this advantage typically comes at the cost of requiring large-scale training data to learn effective visual representations. Although the Transformer has achieved success in vision tasks, its powerful global focus ability reduces the ability to express details. Fine-grained high-frequency details may be weakened through layers of self-attention, thereby damaging the sensitivity to subtle differences. A variety of optimization paths have been explored: replacing self-attention with pure MLPs or simplified token-mixing operators [23], compressing token counts via token selection, pruning, or hierarchical downsampling [2] and introducing bidirectional cross-attention, cross-layer attention, or frequency-domain modeling to better balance low-frequency semantics and high-frequency textures [24,25]. Ref. [26] proposes a new hybrid learning framework that combines dynamic physical models with data-driven quadratic neural networks and bidirectional LSTM. Compared with traditional models, it significantly improves interpretability, credibility and reliability. Ref. [27] proposes a SimAM-Point module, which successfully applies a neuroscience-inspired 3D parameter-free attention mechanism to point cloud feature extraction. By optimizing an energy function, it achieves channel-wise and spatial attention computation without introducing additional parameters, significantly enhancing feature discriminability while maintaining computational efficiency. Nevertheless, most of these solutions do not explicitly model high-frequency information, or they struggle to achieve an ideal trade-off between detail preservation and computational efficiency.

## 3. Materials and Methods

**Figure 2:**
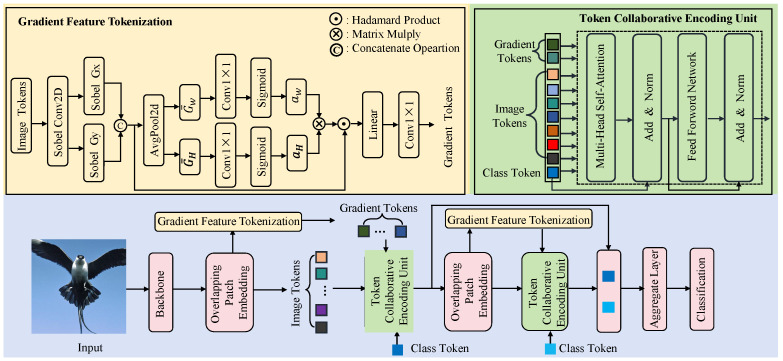
Overview of the proposed Gradient-Aware Spatial Attention for Fine-Grained Image Recognition. The lower part shows the network pipeline. The upper part is divided into two sections: the left section highlights the Gradient Feature Extraction module, while the right section illustrates the Token Collaborative Encoding Unit.

Figure 2 illustrates the overall architecture of our gradient-aware spatial attention framework for fine-grained recognition. An input image is first encoded by a backbone into feature maps and then converted into overlapping patch embeddings to produce spatially contiguous image tokens. To recover high-frequency cues degraded in fine-grained tasks, we propose the Gradient Feature Tokenization (GFT) module: Sobel filters compute per-token gradient magnitude and orientation maps; learnable one-dimensional directional attention is applied along height and width; the two directional weights are combined via the Hadamard product to form a 2D spatial weighting map, which is used to weight local gradient features and project them into gradient tokens aligned with the image-token dimensionality. Image tokens, gradient tokens, and a learnable class token are fed into the Token Collaborative Encoding Unit (TCEU), where multi-head attention enables cross-token interaction to enhance fine-detail representation. The TCEU runs in two stages: stage one refines the initial tokens; stage two introduces a new class token, concatenates it with the stage-one outputs and recomputes gradient tokens, and applies attention for further refinement. Finally, the class tokens from both stages are fused in the Aggregation Layer to produce the final prediction.

### 3.1. Image Feature Tokenization

To convert a two-dimensional image into a sequence suitable for a Transformer, we start from the input image $I\in R^{H\times W\times 3}$, where *H* and *W* denote the image spatial dimensions and the last dimension corresponds to the three RGB channels. A backbone convolutional network F(·) is first used to extract intermediate feature maps:

(1) M=F(I)

where $M\in R^{B\times C^{\prime }\times H^{\prime }\times W^{\prime }}$ denotes the feature map, *B* is the batch size, $C^{\prime }$ is the number of channels, and $H^{\prime }$, $W^{\prime }$ are the spatial resolutions of the feature map. The initial feature extraction stage of the backbone employs three consecutive convolutional layers followed by Mobile Inverted Bottleneck Convolution blocks with squeeze-and-excitation modules [28].

To convert the continuous feature map *M* into image tokens, we adopt the Overlapping Patch Embedding operator E(·) [29]. This operator reduces spatial dimensions via convolutional layers with padding and projects local regions into a high-dimensional embedding space, yielding a sequence of visual tokens:

(2) $$Y=E\left(M\right)\in R^{B\times N\times C}$$

where $N=H^{\prime }\times W^{\prime }$ is the total number of image tokens and *C* denotes the embedding dimension. This design preserves fine-grained local information while facilitating attention-based modeling in subsequent Transformer layers.

### 3.2. Gradient Feature Tokenization

#### 3.2.1. Gradient Feature Extraction

To address the vulnerability of vision Transformers to gradient-feature degradation in fine-grained tasks, we propose the Gradient Feature Tokenization module to enhance fine-grained representation. Built on top of the overlapping patch embedding, this method introduces explicit gradient extraction, directional attention modeling, and projects the weighted gradient features back into token space as augmented inputs to the Transformer. The procedure is as follows.

Let the output of the overlapping patch embedding be $Y\in R^{B\times N\times C}$, where *B* is the batch size, $N=H^{\prime }\times W^{\prime }$ is the sequence length and *C* is the channel dimension. First, reshape *Y* to spatial form $Y^{\prime }\in R^{B\times C\times H^{\prime }\times W^{\prime }}$.

To capture per-channel local directional gradient information, we apply Sobel operators via convolution along the horizontal and vertical directions. Define the convolution kernels as

(3) $$K_{x}=\left[\begin{array}{ccc} 1 & 0 & -1\\ 2 & 0 & -2\\ 1 & 0 & -1 \end{array}\right],\;K_{y}=K_{x}^{⊤}$$

Using grouped convolution (number of groups =C, padding =1), we convolve $Y^{\prime }$ to obtain the horizontal and vertical gradient maps:

(4) $$G_{x}=\mathrm{Conv}2d\left(Y^{\prime };K_{x}\right)\in R^{B\times C\times H^{\prime }\times W^{\prime }}$$

(5) $$G_{y}=\mathrm{Conv}2d\left(Y^{\prime };K_{y}\right)\in R^{B\times C\times H^{\prime }\times W^{\prime }}$$

Concatenating $G_{x}$ and $G_{y}$ yields the joint gradient representation:

(6) $$G=\mathrm{Concat}\left(G_{x},G_{y}\right)\in R^{B\times 2C\times H^{\prime }\times W^{\prime }}\;$$

#### 3.2.2. Directional Attention Construction

To further mine global spatial response patterns of the gradient features, we perform adaptive average pooling along the width and height axes to obtain orthogonal global statistics:

(7) $${\bar{G}}_{H}=\mathrm{AvgPool}2d\left(G\right)\in R^{B\times 2C\times H^{\prime }}$$

(8) $${\bar{G}}_{W}=\mathrm{AvgPool}2d\left(G\right)\in R^{B\times 2C\times W^{\prime }}$$

We apply one-dimensional convolutions (kernel size =3) to ${\bar{G}}_{H}$ and ${\bar{G}}_{W}$, followed by a Sigmoid activation to produce directional attention weights:

(9) $$a_{H}=\sigma \left(\mathrm{Conv}1d({\bar{G}}_{H})\right)\in {\left(0,1\right)}^{B\times 2C\times H^{\prime }}$$

(10) $$a_{W}=\sigma \left(\mathrm{Conv}1d({\bar{G}}_{W})\right)\in {\left(0,1\right)}^{B\times 2C\times W^{\prime }}$$

where σ(·) denotes the Sigmoid function.

#### 3.2.3. Attention Reconstruction and Gradient Weighting

The directional attentions $a_{H}$ and $a_{W}$ are reconstructed into a two-dimensional spatial attention map via outer product:

(11) $$A=a_{H}⊗a_{W}=a_{H}\cdot a_{W}^{⊤}\in R^{B\times 2C\times H^{\prime }\times W^{\prime }}$$

The attention map *A* is applied to the original gradient features *G* element-wise to realize spatially adaptive enhancement:

(12) $$\hat{G}=G⊙A\in R^{B\times 2C\times H^{\prime }\times W^{\prime }}\;$$

where ⊙ denotes the Hadamard product. This process emphasizes discriminative edges and texture regions, improving fine-detail representation.

#### 3.2.4. Gradient Token Generation

Flatten the weighted gradient features $\hat{G}$ into a sequential representation:

(13) $${\hat{G}}_{\_}=\mathrm{Flatten}\left(\hat{G}\right)\in R^{B\times N\times 2C}\;$$

To align with the dimensionality of the original image tokens, apply a linear projection to each spatial position’s 2C-dimensional gradient vector:

(14) $$T_{g}=\mathrm{Linear}\left({\hat{G}}_{\_}\right)\in R^{B\times N\times C}$$

A lightweight convolutional layer is then used to refine channel distribution, yielding the final gradient tokens:

(15) $${\tilde{T}}_{g}=\mathrm{Conv}1d\left(T_{g}\right)\in R^{B\times N\times C}$$

The resulting augmented output serves as input to the Token Collaborative Encoding Unit and participates in self-attention and feed-forward computations. By explicitly modeling gradient information and directional attention and integrating them as token embeddings into the vision Transformer, this mechanism refines sensitivity to local spatial structure and strengthens representations of edges and textures.

### 3.3. Token Collaborative Encoding Unit

To enable effective discrimination between visually similar categories, our model integrates complementary information from multiple representations: the global semantics from a class token, local details from image patches, and structural cues from gradients. To this end, we introduce the Token Collaborative Encoding (TCE) Unit. The TCE unit is designed to fuse these distinct modalities by first concatenating the learnable classification token $x_{\mathrm{class}}$, the gradient-enhanced tokens $x_{\mathrm{gradient}}$ and the original image tokens $x_{\mathrm{image}}$ into a unified sequence. This integrated representation allows the model to collaboratively aggregate high-level semantics with fine-grained structural details for more robust recognition. And it enriches the input sequence in a new complementary form, leveraging the powerful fusion ability of self-attention to achieve a more comprehensive and distinctive representation.

Concretely, the learnable classification token $x_{\mathrm{class}}$, the gradient-enhanced tokens $x_{\mathrm{gradient}}$ and the original image tokens $x_{\mathrm{image}}$ are concatenated to form the initial input:

(16) $$x_{0}=\left[x_{\mathrm{class}};x_{\mathrm{gradient}};x_{\mathrm{image}}\right]$$

The fused sequence is then fed into *L* standard Transformer layers, each comprising multi-head self-attention (MHSA) and a position-wise feed-forward network (MLP). To ensure stable gradient flow and accelerate convergence, pre-normalization with Layer Normalization (LN) [30] is applied before the attention and feed-forward sublayers. Specifically, the computation within each layer is:

(17) $$\begin{array}{cc} x^{\prime } & =\mathrm{MHSA}\left(\mathrm{LN}(x_{\mathrm{in}})\right)+x_{\mathrm{in}},\\ x_{\mathrm{out}} & =\mathrm{MLP}\left(\mathrm{LN}(x^{\prime })\right)+x^{\prime } \end{array}$$

The sequence is projected to query, key and value matrices $Q,K,V\in R^{(M^{2}+O)\times d}$, where $M^{2}$ denotes the number of image tokens and O=1+G indicates one class token plus *G* gradient tokens. The self-attention operation incorporates a relative positional bias $B^{\prime }\in R^{\left(M^{2}+O\right)\times \left(M^{2}+O\right)}$ to encode spatial relationships [23]:

(18) $$\mathrm{Attention}\left(Q,K,V\right)=\mathrm{Softmax}\;\left(\frac{QK^{⊤}}{\sqrt{d}}+B^{\prime }\right)V$$

By jointly fusing semantic, gradient and appearance cues, the Token Collaborative Encoding Unit strengthens the model’s ability to focus on discriminative features, which is particularly important for fine-grained image recognition.

The interaction between the gradient tokens and the class token is not mediated by an explicit, standalone cross-attention mechanism. Instead, their interaction is facilitated implicitly and dynamically within the Token Collaborative Encoding (TCE) Unit through a standard multi-head self-attention (MSA) layer. After concatenating all tokens into a unified sequence, the MSA operation allows every token to attend to every other token. In this process, the class token can selectively attend to and assimilate informative structural cues from the gradient tokens, while the gradient tokens also receive global contextual guidance from the class token.

## 4. Results and Discussion

### 4.1. Datasets

We evaluate the proposed method on four widely used fine-grained visual recognition benchmark datasets (see Table 1): iNaturalist 2018 [31], CUB-200-2011 [32], NABirds [33] and Stanford Cars [34]. iNaturalist 2018 contains millions of real-world images covering plants, animals and fungi, and poses significant challenges due to severe class imbalance and domain shifts. CUB-200-2011 targets bird species recognition and exhibits high inter-class similarity as well as substantial intra-class variations caused by illumination, pose and viewpoint changes. NABirds extends CUB-200-2011 with a hierarchical taxonomy and adds additional difficulty through seasonal appearance changes. Stanford Cars focuses on fine-grained vehicle model recognition, where subtle visual differences among hundreds of car models demand strong discriminative capability. For all datasets, we strictly follow the official training/testing splits in our experiments.

### 4.2. Experimental Details

The backbone network was initialized with weights pretrained on iNaturalist 2021. All input images were resized to 384×384 pixels. Experiments were implemented in PyTorch 2.0 and run on two NVIDIA RTX 4090 GPUs. We optimize using AdamW with a weight decay of 0.05, and employ a cosine annealing learning-rate schedule. Initial learning rates were chosen per-dataset based on preliminary validation: $5\times 10^{-5}$ for iNaturalist 2018, CUB-200-2011 and NABirds, and $5\times 10^{-3}$ for Stanford Cars. To stabilize early training we use a linear warm-up: CUB-200-2011 employs a 20-epoch warm-up whereas the other datasets use a 5-epoch warm-up. Standard data augmentations are applied (random crop, horizontal flip, color jitter), and stochastic depth regularization is used with a maximum drop rate of 0.1. Models were trained for 300 epochs with a batch size of 16. The experiments were conducted in a distributed data-parallel setting using PyTorch’s DistributedDataParallel. To ensure both reproducibility and proper data diversity across training processes, we employed a seed-setting strategy that combines a global base seed with the unique rank of each process. These settings were selected to improve generalization and mitigate overfitting while accommodating dataset-specific characteristics.

### 4.3. Comparison with SOTA Methods

#### 4.3.1. iNaturalist 2018

Table 2 compares our gradient-aware spatial attention network with representative methods on iNaturalist 2018. Unlike MetaFormer [3], which enhances semantic discrimination via joint spatio-temporal tokens, our approach explicitly encodes high-frequency details as gradient tokens and fuses them with class and spatio-temporal tokens using cross-modal attention. This explicit modeling improves localization of fine-grained cues and suppresses background noise. On iNaturalist 2018, our method attains a Top-1 accuracy of 89.9%, demonstrating the benefit of incorporating gradient information.

#### 4.3.2. CUB-200-2011

As shown in Table 3, our gradient-aware spatial attention network attains 92.9% accuracy on CUB-200-2011, outperforming TransFG [47] by over 1.2%. Rather than relying on auxiliary metadata as in MetaFormer [3] or on independent region heads that may break spatial consistency (e.g., IELT [11]), we introduce the Gradient Feature Tokenization (GFT) module to embed gradient-derived high-frequency cues into token embeddings. This explicit gradient modeling improves sensitivity to subtle texture differences and yields substantial gains in fine-grained recognition.

#### 4.3.3. NABirds

As shown in Table 4, the proposed gradient-aware spatial attention fine-grained recognition model achieves a Top-1 accuracy of 93.1% on the NABirds dataset, substantially outperforming TransIFC [47] which attains 91.0%. Unlike TransIFC, which focuses solely on discriminative regions and may overlook important contextual information, our model retains the complete image token sequence while incorporating gradient information, enabling the model to obtain richer feature representations. Compared to MPSA [5], which employs a local region sampling strategy, the proposed method integrates gradient-based features into the image feature maps, thereby balancing global structural cues and local fine-grained details and yielding superior performance.

#### 4.3.4. Stanford Cars

Table 5 reports results on Stanford Cars, where our method achieves a Top-1 accuracy of 95.1%. To better capture fine-grained textures (e.g., body finishes, emblems, and minor ornaments), we propose the Gradient Feature Tokenization (GFT) module, which encodes gradient-derived high-frequency cues into tokens aligned with image embeddings. The GFT preserves spatial structure while strengthening discriminative detail and yields consistent gains in challenging fine-grained scenarios.

### 4.4. Visualization Analysis

**Figure 3:**
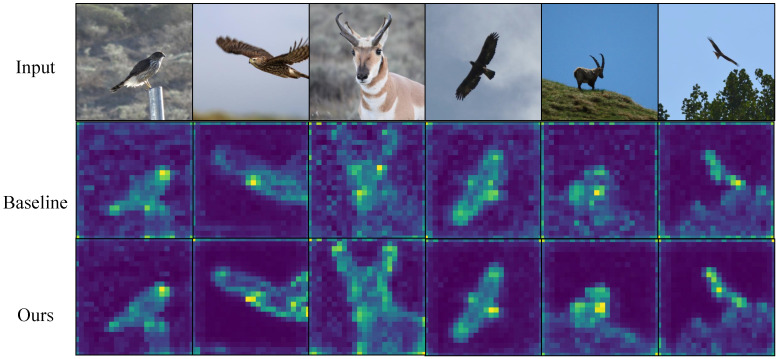
Visualization on iNaturalist 2018. Our method emphasizes species-specific cues despite large intra-class diversity and clutter.

**Figure 4:**
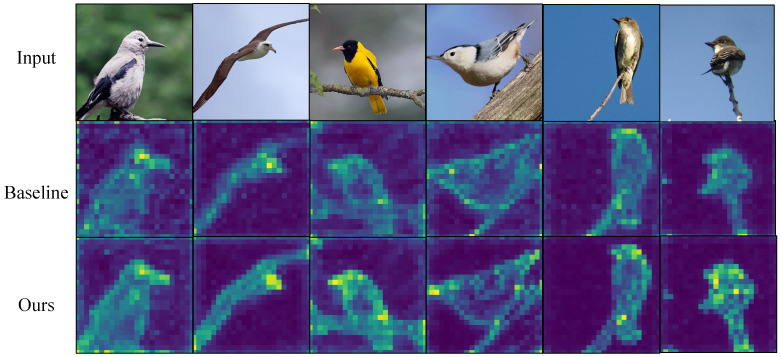
Visualization on NABirds. Compared with the baseline, our model keeps stable attention on birds across complex scenes and poses.

**Figure 5:**
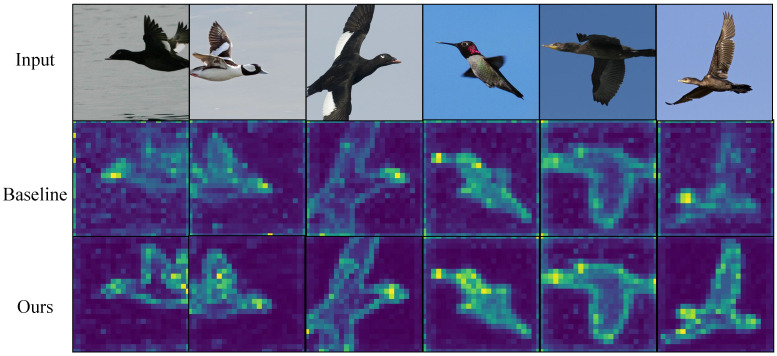
Visualization on CUB-200-2011. Our method focuses on key body parts (e.g., head, wings) while reducing background distraction.

**Figure 6:**
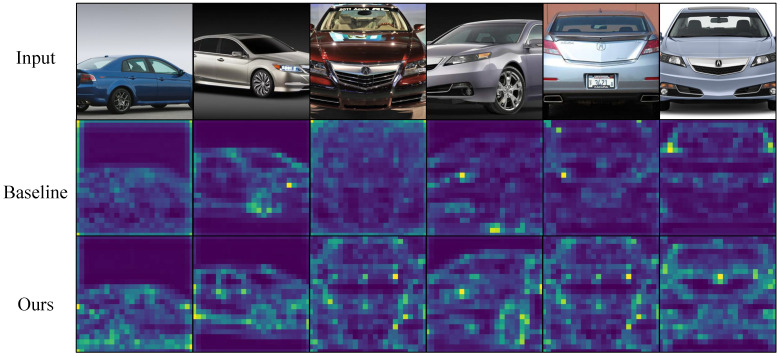
Visualization on Stanford Cars. The model consistently attends to key components (e.g., headlights, wheels), capturing fine shape differences.

Figure 3, Figure 4, Figure 5 and Figure 6 present attention maps on iNaturalist 2018, CUB-200-2011, NABirds and Stanford Cars. The first row shows input images, the second row shows maps from the comparative model [3], and the third row shows maps from our gradient-aware spatial attention. While the comparative model attends to background or dispersed regions, our method concentrates on foreground edges, textures and discriminative parts. These results indicate that gradient tokens supply effective high-frequency cues, improving localization and discrimination of subtle features.

### 4.5. Ablation Study

#### 4.5.1. Effectiveness of the Proposed Module

The core component of the proposed gradient-aware spatial attention framework for fine-grained image recognition is the Gradient Feature Tokenization module. This module explicitly encodes image gradient cues—such as edges and subtle textures—into gradient tokens to enhance standard visual representations.

As shown in Table 6, integrating gradient tokens consistently improves performance across multiple fine-grained recognition datasets. On the iNaturalist 2018 dataset, performance increases by 1.2%, demonstrating the effectiveness of gradient-aware modeling in complex real-world biodiversity scenarios characterized by significant intra-class variation. Notably, on the challenging NABirds dataset, our method achieves state-of-the-art performance, surpassing previous leading approaches. These results indicate that the Gradient Feature Tokenization module not only provides complementary high-frequency structural cues but also exhibits strong generalization ability in diverse and realistic fine-grained recognition tasks.

#### 4.5.2. Impact of the Number of Gradient Tokens

As shown in Table 7, we investigate the effect of varying the number of gradient tokens in the Gradient Feature Tokenization module on model performance. On the CUB-200-2011 dataset, the best performance is achieved when the number of gradient tokens is set to 12, yielding the optimal balance between accuracy and efficiency. Therefore, this configuration is adopted as the default setting. Similar trends are observed across other datasets, indicating that using 12 gradient tokens provides stable and generalizable performance in diverse fine-grained image recognition tasks.

#### 4.5.3. Analysis of Hard Cases

**Figure 7:**
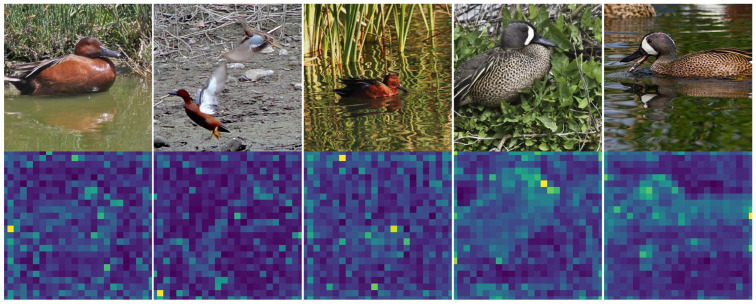
Hard cases on NABirds dataset.

To thoroughly evaluate the robustness and failure modes of our model, we conducted systematic analysis and visualization of attention features on hard cases. As shown in Figure 7, our analysis reveals that these challenging samples primarily exhibit two typical patterns: (1) severe occlusion of critical discriminative regions (e.g., bird heads obscured by foliage), preventing the model from capturing decisive features; (2) strong semantic interference from background elements, causing attentional drift in the model. These findings reveal inherent limitations in the current gradient-aware mechanism. Future work will explore multimodal feature fusion to address a broader range of challenging cases.

#### 4.5.4. Model Complexity Analysis

As shown in Table 8, to further validate the efficiency of our model, we compare its computational cost with several recent works. Our model requires 52.7 G FLOPs, which is notably lower than most contemporary models. In terms of model capacity, the number of the parameters of our method is 88.1 M, which is slightly higher than that of MetaFormer. Overall, these results demonstrate a superior trade-off between performance and computational complexity.

#### 4.5.5. Sensitivity Analysis of Sobel Kernel Size

To determine the optimal configuration for the gradient-aware module, we conducted a sensitivity analysis on the size of the Sobel operator. The results, presented in Table 9, compare the performance achieved with kernel sizes of 3×3, 5×5, and 7×7. 3×3 kernel is observed to yield the best performance on the benchmark. We posit that the smaller kernel excels at capturing the fine-grained, high-frequency details most pertinent to our task. Conversely, larger kernels, while offering a broader receptive field, may over-smooth these localized and critical features, leading to a discernible performance drop.

## 5. Conclusions

To address the insufficient exploitation of high-frequency details and the lack of effective local–global integration in fine-grained image recognition, this paper proposes a gradient-aware spatial attention framework. By explicitly modeling gradient signals, the method constructs a gradient-aware spatial attention mechanism that enables collaborative modeling of local discriminative details and global semantics. Experimental results demonstrate that the proposed approach significantly improves accuracy and robustness across multiple benchmark fine-grained datasets, particularly under challenging conditions such as background clutter and limited training samples. This work explores a novel pathway for efficiently incorporating explicit visual priors into Transformer architectures, enhancing the model’s sensitivity to subtle structural differences and contributing to the interpretability of vision Transformers. The proposed paradigm provides valuable insights for future research on multi-scale local prior modeling and hybrid attention mechanism design. In the future, integrating classic high-frequency detail enhancement techniques, such as Laplacian filtering, wavelet and dedicated edge enhancement modules, will be an important direction for our future research.

Furthermore, we point out that the method’s performance may be challenged in scenarios with: (1) extremely low-resolution inputs where gradient information becomes unreliable, (2) cases where critical discriminative features reside in color or spectral properties rather than structural patterns, and (3) situations with severe occlusion that obscures most structural details. Systematically studying these boundary conditions through more challenging test cases represents an important direction for future work.

The proposed gradient-aware spatial attention framework, validated on fine-grained image recognition tasks, exhibits substantial potential for various critical application domains requiring discrimination of subtle structural differences. In medical imaging, this approach can enhance the identification of fine edges and texture patterns, as well as improve recognition of pathological cellular structures in histopathological slides. Similarly, for industrial automated inspection, the framework can be deployed to detect microscopic defects such as hairline cracks on metal surfaces, micro-scratches on optical lenses, or faint anomalies in textiles. Adapting our framework to these domains through retraining on domain-specific data represents a straightforward and productive direction for future research.

## Figures and Tables

**Table 1:** The Details of Datasets We Used and the Corresponding Learning Rates.

Dataset	Class	Train	Test	LR
iNaturalist 2018 [31]	8142	437,513	24,426	5 $\times \;10^{-5}$
CUB-200-2011 [32]	200	5994	5794	5 $\times \;10^{-5}$
NABirds [33]	555	23,929	24,633	5 $\times \;10^{-5}$
Stanford Cars [34]	196	8114	8041	5 $\times \;10^{-3}$

**Table 2:** Comparison Results on iNaturalist 2018.

Method	Publication	Accuracy (%)
BS-CMO [35]	CVPR 2022	74.0
ViT-L [36]	ECCV 2022	75.3
GPaCo [37]	IEEE 2023	75.4
MixMIM-B [38]	CVPR 2023	77.5
Presence-Only [39]	ICCV 2019	77.5
CaiT-M-36U224 [40]	ICCV 2021	78.0
MixMIM-L [38]	CVPR 2023	80.3
VL-LTR [41]	ECCV 2022	81.0
RDNet-L [42]	ECCV 2024	81.8
OMNIVORE [43]	CVPR 2022	84.1
SWAG [44]	CVPR 2022	86.0
MAE [45]	CVPR 2022	86.8
Hiera-H [46]	PMLR 2023	87.3
MetaFormer [3]	arXiv 2022	88.7
Ours	–	**89.9**

**Table 3:** Comparison Results on CUB-200-2011.

Method	Publication	Accuracy (%)
DenseNet161 [48]	CVPR 2017	85.5
PMG [49]	CVPR 2023	90.6
CP-CNN [50]	TIP 2022	91.4
LGTF [4]	ICCV 2023	91.5
TransIFC [47]	TMM 2023	91.0
CAMF [51]	SPL 2021	91.2
RAMS-Trans [52]	AAAI 2021	91.3
PMRC [53]	CVPR 2023	91.5
ViT-Net [54]	ICML 2022	91.6
TransFG [2]	AAAI 2022	91.7
IELT [11]	TMM 2023	91.8
MP-FGVR [55]	AAAI 2024	91.8
ACC-VIT [56]	AAAI 2024	91.8
DACL [24]	CVPR 2022	92.0
MetaFormer [3]	arXiv 2022	92.5
CGL [57]	TMM 2025	92.6
MPSA [5]	TIP 2024	92.8
Ours	–	**92.9**

**Table 4:** Comparison Results on NABirds.

Method	Publication	Accuracy (%)
DenseNet161 [48]	CVPR 2017	83.1
GaRD [58]	CVPR 2021	88.0
SEB [59]	TPAMI 2023	88.2
PMG-V2 [60]	TPAMI 2022	88.4
GDSPM-Net [61]	PR 2023	89.0
LGTF [4]	ICCV 2023	90.4
TransFG [2]	AAAI 2022	90.8
IELT [11]	TMM 2023	90.8
TransIFC [47]	TMM 2023	90.9
MP-FGVR [55]	AAAI 2024	91.0
ACC-ViT [56]	PR 2024	91.4
CGL [57]	TMM 2025	91.7
MPSA [5]	TIP 2024	92.5
MetaFormer [3]	arXiv 2022	92.8
Ours	–	**93.1**

**Table 5:** Comparison Results on Stanford Cars.

Method	Publication	Accuracy (%)
LDH-ViT [62]	PR 2025	93.1
DATL [63]	ISVC 2020	94.5
RsNet50-ECC [64]	TNNLS 2024	94.7
TransFG [2]	AAAI 2022	94.8
ViT-Net [54]	ICML 2022	95.0
MetaFormer [3]	arXiv 2022	95.0
Ours	–	**95.1**

**Table 6:** Ablation Studies of the Proposed Gradient Feature Tokenization Modules.

GFE	iNaturalist 2018	CUB-200-2011	NABirds	Stanford Cars
-	88.70	92.50	92.80	94.98
✓	**90.46**	**92.86**	**93.23**	**95.27**

**Table 7:** Effect of gradient token count on Gradient-Aware Spatial Attention for Fine-Grained Image Recognition accuracy.

Token_Numbers	0	4	8	12	16
Accuracy (%)	92.50	92.78	92.80	92.87	92.82

**Table 8:** Comparison of the properties of the proposed model with the existing state-of-the-art models.

Models	Param (M)	FLOPs (G)
TransFG [2]	86.4	130.2
IELT [11]	93.5	73.2
ACC-ViT [56]	87.0	162.9
Swin-Base [23]	87.1	47.2
ViT-Net [54]	92.2	65.6
MPSA [60]	98.5	67.9
PMRC (SwinTransformer) [53]	89.0	-
PMRC (VGG16) [53]	139.0	-
MetaFormer [65]	81.0	49.7
Ours	88.1	52.7

**Table 9:** Sensitivity Analysis of Sobel Kernel Size on Nabirds dataset.

Sobel Kernel Size	3×3	5×5	7×7
Accuracy (%)	93.1	93.07	93.03

## Data Availability

The source code is available at: https://github.com/bingbeu/GASA.git (accessed on 10 December 2025).
